# DEVELOPING A TOUCHSCREEN COMMUNICATION DEVICE TO EMPOWER PEOPLE WITH DEMENTIA AND THEIR CAREGIVERS

**DOI:** 10.1093/geroni/igad104.1756

**Published:** 2023-12-21

**Authors:** Ellen Brown, Nicole Ruggiano, Sai Allala, Gabriel Puche, Lisa Roberts, C Framil, Mariateresa Munoz, Monica Hough

**Affiliations:** Nicole Wertheim College of Nursing and Health Sciences, Florida International University, Miami, Florida, United States; University of Alabama, Hoover, Alabama, United States; Florida International University, Miami, Florida, United States; Florida International University, Miami, Florida, United States; Florida International University, Miami, Florida, United States; Florida International University, Miami, Florida, United States; Florida International University, Miami, Florida, United States; Florida International University, Miami, Florida, United States

## Abstract

People living with dementia (PLWD) often experience communication deficits that create frustration and challenges to caregiving and clinical care for this population. It also constrains person-centered care. To address communication challenges, an interdisciplinary team developed a mobile communication app that promotes communication by PLWDs about their daily care preferences and experiences. This app, My Person Assisted Touchscreen Interface (My PATI), empowers PLWDs and their caregivers by supporting communication with a fully-customizable platform. This platform allows the PLWD to express preferences for daily activities, such as preferred foods and clothing options. It also enables the PLWD to express their experiences relevant to symptoms of pain, depression, and sleep quality. This presentation will describe a multi-phase Quality Implementation Framework that the research team has applied in developing My PATI to maximize its potential for being adopted into the home and residential care settings and used by multiple caregivers. The presentation will include the My PATI implementation video and foundational research: qualitative interviews with PLWDs, caregivers of PLWD, alpha and beta testing with dyads of caregivers and PLWDs, and extensive assessment and development planning with clinicians, geriatric researchers, computer scientists, and a graphic artist to inform My PATI functions and design. The presentation will also describe the designs of future pilot testing and randomized control trial studies that are planned for 2024.

